# Rituximab and new regimens for indolent lymphoma: a brief update from 2012 ASCO Annual Meeting

**DOI:** 10.1186/1475-2867-12-38

**Published:** 2012-08-23

**Authors:** Jiangning Zhao, Zhenshu Xu, Delong Liu, Quanyi Lu

**Affiliations:** 1 Department of Hematology, Zhongshan Hospital of Xiamen University, Xiamen 361004, China; 2 Institute of Hematology, Fujian Union Hospital, Fuzhou, China; 3 Division of Hematology and Oncology, New York Medical College and Westchester Medical Center, Valhalla, NY 10595, USA

## Abstract

Indolent lymphoma (IL), the second most common lymphoma, remains incurable with chemotherapy alone. While R-CHOP (rituximab, cyclophosphamide, doxorubicin, vincristine, prednisone) remains the standard frontline regimen for diffuse Large B –cell lymphoma, the optimal chemotherapy regimen for frontline therapy of advanced IL remains uncertain. FCR (fludarabine, cyclophosphamide, rituximab) has been shown to be better than fludarabine alone and fludarabine plus cyclophosphamide for IL. In FOLL05 trial, R-CHOP was compared with R-CVP (cyclophosphamide, vincristine, prednisone) and R-FM (fludarabine, mitoxantrone). The study showed that R-CHOP appears to have the best risk-benefit ratio for IL. The StiL NHL1 trial showed that BR (bendamustine, rituximab) has longer progression free survival and is better tolerated than R-CHOP. Long-term complications with secondary malignancies between the two regimens appear to be comparable. In this review, new combination regimens reported at 2012 ASCO annual meeting were evaluated for frontline and salvage therapy of indolent lymphoma.

## 

Rituximab has essentially changed the natural history of diffuse Large B –cell lymphoma (DLBCL) [1-7]. R-CHOP (rituximab, cyclophosphamide, doxorubicin, vincristine, prednisone) remains the standard frontline regimen for DLBCL [8-11]. New agents and biomarkers are being studied in an attempt to improve upon the current efficacy of R-CHOP [12-19]. Meanwhile, indolent lymphoma, the second most common subtype of lymphoma, remains incurable with chemotherapy alone [20-23]. FCR (fludarabine, cyclophosphamide, rituximab) has been shown to be better than fludarabine alone and fludarabine plus cyclophosphamide for untreated chronic lymphoid leukemia (CLL) [24-26]. Another regimen, PCR (pentostatin, cyclophosphamide, rituximab), was found to be comparable to FCR for untreated CLL[21]. In this review, new combination regimens reported at 2012 ASCO annual meeting were evaluated for frontline and salvage therapy of indolent lymphoma.

## Rituximab for low burden indolent lymphoma

For low burden indolent lymphoma (LBIL), the standard approach is still “watch & wait”. A randomized study was conducted for low burden IL, the E4402 RESORT study [27]. The hypothesis was that rituximab (R) treatment of LBIL and maintenance rituximab (MR) would be superior to rituximab retreatment at disease progression. LBIL (small lymphocytic lymphoma, MZL, FL) were treated with R weekly x 4, then responders were randomized to receive MR (R x 1 q 3 months until progression), or RR (R weekly x 4 at progression). The primary endpoint was time to treatment failure (TTF). At the 2011 ASH meeting update, 384 with FL were enrolled. Overall response (CR + PR) was 71%. These 274 responders were then randomized to MR (n = 140) or RR (n = 134). The median follow-up was 3.8 yrs. There was no significant difference in TTF between the two groups (MR 3.9 yr vs. RR 3.6 yr). At 3 yrs, the rate of time to cytotoxic chemotherapy (TTTC) was 95% for MR vs. 86% for RR patients (p = .027). Therefore, MR and RR were comparable for the primary endpoint, TTF.

The E4402 results on non-FL lymphomas (SLL, MZL) were presented at 2012 ASCO [28]. A total of 137 non-FL patients were enrolled. Overall response (CR + PR) was 41% (n = 57). These 57 patients were then randomized to MR (n = 32) or RR (n = 25). The median follow-up was 4.3 yrs. TTF of MR group was better than that of RR group (MR 3.74 yr vs. RR 1.07 yr, p = 0.0002; HR 4.95). At 3 yrs, the rate of freedom from cytotoxic treatment was 100% for MR vs. 70% for RR patients (p = 0.0002). There were 2 patients with grade III-IV toxicity (1 neutropenia, 1 encephalopathy) in MR group. In conclusion, this planed subgroup analysis for non-FL patients revealed significant benefits in TTF and TTTC.

These results in non-FL differ from those of FL patients in this trial. The overall response rate to R induction was higher in FL patients (FL 71% vs. non-FL 41%; p < .0001), but no TTF benefit was observed with MR for FL patients.

## Rituximab: study of pharmacokinetics (PK) and pharmacodynamics (PD) for faster infusion

Rituximab (R) is typically infused over 4–6 hours. Faster infusion can improve patient’s convenience and optimize utilization of oncology unit resources. A prospective, open-label, multicenter, single-arm trial was conducted to assess the safety, PK and PD of a 90-min infusion rate for R in NHL patients [29]. Patients (≥18 yrs old) with untreated DLBCL or FL received R-chemotherapy at the standard infusion rate (4–6 h) in cycle 1. Further R infusion was given over 90-min to patients who had no severe reactions and had circulating lymphocyte counts ≤ 5000/μl prior to cycle 2. A total of 14 patients who completed all cycles with faster infusion were asked to provide further serum samples up to 16 weeks after the last chemotherapy cycle. PK parameters, including R terminal half-life (t_½_), maximum serum concentration (C_max_), systemic clearance (CL) and volume of distribution (V) were evaluated. A total of 365 patients received the 90-min infusion rate at cycle 2. Peak R levels were higher than seen in published data of q3w R regimens but comparable with levels seen for q1w R regimens. The PK data and B cell (CD19+) depletion were similar between the groups of faster infusion and standard rate. These results suggest that a faster infusion of R has similar safety and PK data to the standard infusion rate. It is however important to evaluate the efficacy and the long-term outcome of the lymphoma treated at the faster infusion rate.

## R-CVP vs R-CHOP vs R-FM: the final analysis of FOLL05 trial

There has been no international consensus on frontline optimal chemotherapy regimen for patients with advanced follicular lymphoma (FL). A final report of FOLL05 trial was presented at 2012 ASCO on a randomized comparison of R-CVP x 8 vs R-CHOP x 6 vs R-FM x 6 [30]. Maintenance therapy was not allowed in this randomized trial. The primary end point was time to treatment failure (TTF) which was defined as failure of induction therapy, progressive or relapse disease and death from any causes. A total of 534 patients were enrolled; 30 were not evaluable. The patients’ characteristics were as the following: median age = 56 years (range 30–75), 63% of patients had stage IV disease, 37% had unfavorable disease (FLIPI score 3–5).

There was no significant difference of overall response rate (CR + PR) for the whole group (91%, p = 0.247). The median follow-up time was 34 months. The 3-year TTF for patients treated with R-CVP, R-CHOP and R-FM was 46%, 64% and 61%, respectively (R-CHOP *vs* R-CVP p = 0.007; R-FM *vs* R-CVP p = 0.021; R-FM *vs* R-CHOP p = 0.969). Therefore, R-CVP had the worst TTF among the three groups.

The 3-year overall survival rate (OS) was not significantly different among the three groups (R-CVP 98%, R-CHOP 95%, and R-FM group 93%). Patients in R-FM group had a higher rate of severe neutropenia (64% *vs* 28% R-CVP, p < 0.001; *vs* 50% R-CHOP, p = 0.015).

At this final analysis, a total of 23 patients developed secondary malignancies (R-CVP 2%, R-CHOP 3% and R-FM 8%), indicating a higher incidence of secondary malignancies in R-FM group.

In summary, R-CHOP appears to be the best among the three with regard to the risk-benefit consideration for frontline therapy of advanced follicular lymphoma. One weakness of the trial was that it used CT instead of more sensitive PET scan to define complete response. In addition, TTF could have been improved if maintenance therapy was included [3,31].

## BR vs R-CHOP: StiL NHL1 trial update

Bendamustine has been used for frontline treatment of CLL and for relapsed /refractory indolent lymphoma [32-36]. However its role in frontline therapy of indolent lymphoma has not been established. BR was compared with R-CHOP in a multicenter, randomized, phase III study for first-line treatment, the StiL NHL1 trial [37]. Patients with newly diagnosed indolent lymphoma and mantle cell lymphoma were enrolled. An updated analysis with a cut-off date for 31 Oct 2011 was presented at 2012 ASCO. The primary endpoint was PFS. Patients received a maximum of 6 cycles after randomization. The study enrolled 549 patients, 514 of them were evaluable (261 BR; 253 R-CHOP). The median age was 64 years.

The median follow-up was 45 months at this update. The PFS of BR was more than doubled in comparison with that of R-CHOP (69.5 versus 31.2 months; HR 0.58, 95% CI 0.44–0.74; p < 0.001). This advantage of BR was seen across all histological subtypes except marginal zone lymphoma. This advantage of BR was true both for patients ≤60 years (n = 199, HR 0.52, P = 0.002), and for patients >60 years (n = 315, HR 0.62, P = 0.002).

This update also reported more results based on risk factors. Compared with R-CHOP, BR significantly prolonged PFS in patients with normal LDH (P < 0.001). The longer PFS with BR was seen in both favorable (0–2 factors, p = 0.043) and unfavorable (3–5 factors, p = 0.068) FLIPI subgroups of patients with follicular lymphoma.

There were 74 treatment failures in the BR group, 116 in the R-CHOP group. In the R-CHOP group, 45% (52/116) received BR as salvage regimen. There was no significant difference in overall survival. Previously there have been unanswered questions regarding long-term complications with bendamustine. At this update, more information was provided in terms of secondary malignancies. With a median follow-up of 45 months, there appears to have no significant differences for secondary malignancies between the two groups (20 in BR, 23 in R-CHOP). In particular, there was one hematological malignancy in each group (1 MDS in BR, 1 AML in R-CHOP).

With this updated analysis, it appears that for newly diagnosed indolent lymphoma and elderly patients with MCL, BR has longer PFS and is better tolerated than R-CHOP. Long-term complications with secondary malignancies appear to be comparable.

## R-CHOP vs CHOP-I^131^ tositumomab

Two agents have been approved for lymphoma radioimmunotherapy[38-45]. SWOG and CALGB intergroup trial, S0016, enrolled 554 patients between 3/1/2001 and 9/15/2008 to compare the safety and efficacy of 2 immunochemotherapy regimens, R-CHOP vs CHOP-I^131^ tositumomab (CHOP-RIT), for untreated patients with bulky stage II, III or IV FL[46]. Patients were randomized to CHOP-R x 6 or CHOP-RIT X 6. Both treatment regimens had excellent outcome with no significant differences (2 yr PFS: CHOP-R 76% vs CHOP-RIT 80%, p =0.11; 2 yr OS: CHOP-R 97% vs CHOP-RIT 93%, p =0.08). The serum-β2M, LDH level, and FLIPI score were found to be the strongest prognostic factors for PFS and OS by multivariable analysis.

## Gemcitabine, rituximab and oxaliplatin (GROC)

Oxaliplatin has been studied for lymphoma therapy in various combination regimens [47-50]. Recently, in the salvage setting for relapsed/refractory NHL, gemcitabine, rituximab and oxaliplatin (GROC) was shown in a phase II trial to have an overall response rate of 58%, grade 3–4 thrombocytopenia of 9% and neutropenic fever of 3.5%[51]. No severe non-hematologic toxicities were observed. Patients received rituximab (375 mg/m^2^) on day 1. On day 2, patients received gemcitabine (1000 mg/m^2^) and oxaliplatin (100 mg/m^2^). This was repeated every two weeks. A total of 58 patients were enrolled. The median age was 72 years (range 24 to 88 years). Median PFS was 134 days (95% CI 115–153) and median OS was 296 days (95% CI 164–428). The risk factors of age, IPI, LDH and albumin level did not influence the responses, but prior rituximab (p = 0.02) and response to initial therapy (p = 0.04) correlated with better outcomes. Therefore, GROC may be a useful salvage regimen for relapsed/refractory NHL. After complete of GROC therapy, 9 patients were successfully mobilized, collected and transplanted.

## Conclusions and future directions

The optimal chemotherapy regimen for frontline therapy of advanced indolent lymphoma remains uncertain. Compared with R-CVP and R-FM, R-CHOP appears to have the best risk-benefit ratio. The StiL NHL1 trial showed that BR has longer PFS and is better tolerated than R-CHOP. Long-term complications with secondary malignancies between the two regimens appear to be comparable. Many novel agents with different mechanisms of action are being explored [52-55]. The Bruton’s tyrosine kinase inhibitors appear to be very active in chronic lymphoid leukemia and refractory lymphoma [56-59]. It would be interesting to see whether adding novel agents to BR or R-CHOP can further improve the outcomes of advanced indolent lymphoma.

## Abbreviations

CR: Complete response; CRu: Complete response unconfirmed; PD: Pharmacodynamics; PK: Pharmacokinetics; PR: Partial response; ORR: Overall response rate; OS: Overall survival; PFS: Progression free survival; SD: Stable disease; TTF: Time to treatment failure; CLL: Chronic lymphoid leukemia; DLBCL: Diffuse Large B –cell lymphoma; FL: Follicular lymphoma; IL: Indolent lymphoma; MCL: Mantle cell lymphoma; MZL: Marginal zone lymphoma; SLL: Small lymphocytic lymphoma; FCR: Fludarabine, cyclophosphamide, rituximab; PCR: Pentostatin, cyclophosphamide, rituximab; R-CHOP: Rituximab, cyclophosphamide, doxorubicin, vincristine, prednisone; R-CVP: Cyclophosphamide, vincristine, prednisone; R-FM: Fludarabine, mitoxantrone.

## Competing interests

Authors have no relevant conflict of interest.

## Authors’ contribution

DL and QL designed the study. All authors participated in data collection and draft preparation. All authors read and approved the final manuscript.

## References

[B1] Harjunpaa A Junnikkala S Meri S Rituximab (anti-CD20) therapy of B-cell lymphomas: direct complement killing is superior to cellular effector mechanisms Scand J Immunol 2000 51 6 634 641 10.1046/j.1365-3083.2000.00745.x 10849376

[B2] Plosker GL Figgitt DP Rituximab: a review of its use in non-Hodgkin's lymphoma and chronic lymphocytic leukaemia Drugs 2003 63 8 803 843 10.2165/00003495-200363080-00005 12662126

[B3] Habermann TM Weller EA Morrison VA Gascoyne RD Cassileth PA Cohn JB Dakhil SR Woda B Fisher RI Peterson BA Rituximab-CHOP versus CHOP alone or with maintenance rituximab in older patients with diffuse large B-cell lymphoma Journal of clinical oncology: official journal of the American Society of Clinical Oncology 2006 24 19 3121 3127 10.1200/JCO.2005.05.1003 16754935

[B4] Coiffier B Rituximab in diffuse large B-cell lymphoma Clinical advances in hematology & oncology: H&O 2004 2 3 156 157 16166943

[B5] Coiffier B Rituximab therapy in malignant lymphoma Oncogene 2007 26 25 3603 3613 10.1038/sj.onc.1210376 17530014

[B6] Coiffier B Haioun C Ketterer N Engert A Tilly H Ma D Johnson P Lister A Feuring-Buske M Radford JA Rituximab (anti-CD20 monoclonal antibody) for the treatment of patients with relapsing or refractory aggressive lymphoma: a multicenter phase II study Blood 1998 92 6 1927 1932 9731049

[B7] Coiffier B Pfreundschuh M Stahel R Vose J Zinzani PL Aggressive lymphoma: improving treatment outcome with rituximab Anti-cancer drugs 2002 13 Suppl 2 S43 S50 12710590 10.1097/00001813-200211002-00007

[B8] Pfreundschuh M Trumper L Osterborg A Pettengell R Trneny M Imrie K Ma D Gill D Walewski J Zinzani P CHOP-like chemotherapy plus rituximab versus CHOP-like chemotherapy alone in young patients with good-prognosis diffuse large-B-cell lymphoma: a randomised controlled trial by the MabThera International Trial (MInT) Group Lancet Oncol 2006 7 5 379 391 10.1016/S1470-2045(06)70664-7 16648042

[B9] Coiffier B Lepage E Briere J Herbrecht R Tilly H Bouabdallah R Morel P Van Den Neste E Salles G Gaulard P CHOP chemotherapy plus rituximab compared with CHOP alone in elderly patients with diffuse large-B-cell lymphoma N Eng J Med 2002 346 4 235 242 10.1056/NEJMoa011795 11807147

[B10] Coiffier B Rituximab and CHOP-like chemotherapy in good-prognosis diffuse large-B-cell lymphoma Nat Clin Pract Oncol 2006 3 11 594 595 17080173 10.1038/ncponc0638

[B11] Coiffier B Rituximab in combination with CHOP improves survival in elderly patients with aggressive non-Hodgkin's lymphoma Semin Oncol 2002 29 2 Suppl 6 18 22 12040530 10.1053/sonc.2002.32749

[B12] Tilly H Morschhauser F Salles G Casasnovas RO Feugier P Molina TJ Jardin F Terriou L Haioun C Coiffier B Phase 1b study of lenalidomide in combination with rituximab-CHOP (R2-CHOP) in patients with B-cell lymphoma 2012 Leukemia official journal of the Leukemia Society of America, Leukemia Research Fund, UK 10.1038/leu.2012.172 22733106

[B13] Jais JP Haioun C Molina TJ Rickman DS de Reynies A Berger F Gisselbrecht C Briere J Reyes F Gaulard P The expression of 16 genes related to the cell of origin and immune response predicts survival in elderly patients with diffuse large B-cell lymphoma treated with CHOP and rituximab Leukemia: official journal of the Leukemia Society of America, Leukemia Research Fund, UK 2008 22 10 1917 1924 10.1038/leu.2008.188 PMC2675107 18615101

[B14] Wu ZL Song YQ Shi YF Zhu J High nuclear expression of STAT3 is associated with unfavorable prognosis in diffuse large B-cell lymphoma J Hematol Oncol 2011 4 1 31 10.1186/1756-8722-4-31 21806788 PMC3163635

[B15] Johnson P New targets for lymphoma treatment Annals of oncology: official journal of the European Society for Medical Oncology / ESMO 2008 19 Suppl 4) iv56 iv59 18519406 10.1093/annonc/mdn198

[B16] Johnston P Yuan R Cavalli F Witzig T Targeted therapy in lymphoma J Hematol Oncol 2010 3 1 45 10.1186/1756-8722-3-45 21092307 PMC3003620

[B17] Sacco A Issa G Zhang Y Liu Y Maiso P Ghobrial I Roccaro A Epigenetic modifications as key regulators of Waldenstrom's Macroglobulinemia biology J Hematol Oncol 2010 3 1 38 10.1186/1756-8722-3-38 20929526 PMC2964547

[B18] Budhu A Ji J Wang X The clinical potential of microRNAs J Hematol Oncol 2010 3 1 37 10.1186/1756-8722-3-37 20925959 PMC2958878

[B19] Furman RR Martin P Ruan J Cheung YK Vose JM LaCasce AS Elstrom R Coleman M Leonard JP Phase 1 trial of bortezomib plus R-CHOP in previously untreated patients with aggressive non-Hodgkin lymphoma Cancer 2010 116 23 5432 5439 10.1002/cncr.25509 20665890

[B20] Wang J Ke X-Y The Four types of Tregs in malignant lymphomas J Hematol Oncol 2011 4 1 50 10.1186/1756-8722-4-50 22151904 PMC3253040

[B21] Reynolds C Di Bella N Lyons RM Hyman W Richards DA Robbins GJ Vellek M Boehm KA Zhan F Asmar L A Phase III trial of fludarabine, cyclophosphamide, and rituximab vs. pentostatin, cyclophosphamide, and rituximab in B-cell chronic lymphocytic leukemia Invest New Drugs 2012 30 3) 1232 1240 21922186 10.1007/s10637-011-9737-y

[B22] Hornberger J Reyes C Shewade A Lerner S Friedmann M Han L Gutierrez H Satram-Hoang S Keating MJ Cost-effectiveness of adding rituximab to fludarabine and cyclophosphamide for the treatment of previously untreated chronic lymphocytic leukemia Leuk Lymphoma 2012 53 2 225 234 10.3109/10428194.2011.605918 21824050

[B23] Tedeschi A Benevolo G Varettoni M Battista ML Zinzani PL Visco C Meneghini V Pioltelli P Sacchi S Ricci F Fludarabine plus cyclophosphamide and rituximab in Waldenstrom macroglobulinemia: an effective but myelosuppressive regimen to be offered to patients with advanced disease Cancer 2012 118 2 434 443 10.1002/cncr.26303 21732338

[B24] Zhou Y Tang G Medeiros LJ McDonnell TJ Keating MJ Wierda WG Wang SA Therapy-related myeloid neoplasms following fludarabine, cyclophosphamide, and rituximab (FCR) treatment in patients with chronic lymphocytic leukemia/small lymphocytic lymphoma Modern pathology: an official journal of the United States and Canadian Academy of Pathology, Inc 2012 25 2 237 245 10.1038/modpathol.2011.158 PMC11753509 22080061

[B25] Keating MJ O'Brien S Albitar M Lerner S Plunkett W Giles F Andreeff M Cortes J Faderl S Thomas D Early results of a chemoimmunotherapy regimen of fludarabine, cyclophosphamide, and rituximab as initial therapy for chronic lymphocytic leukemia Journal of clinical oncology: official journal of the American Society of Clinical Oncology 2005 23 18 4079 4088 10.1200/JCO.2005.12.051 15767648

[B26] Hallek M Fischer K Fingerle-Rowson G Fink AM Busch R Mayer J Hensel M Hopfinger G Hess G von Grunhagen U Addition of rituximab to fludarabine and cyclophosphamide in patients with chronic lymphocytic leukaemia: a randomised, open-label, phase 3 trial Lancet 2010 376 9747 1164 1174 10.1016/S0140-6736(10)61381-5 20888994

[B27] Kahl BS Hong F Williams ME Gascoyne RD Wagner LI Krauss JC Horning SJ Results of Eastern Cooperative Oncology Group Protocol E4402 (RESORT): A Randomized Phase III Study Comparing Two Different Rituximab Dosing Strategies for Low Tumor Burden Follicular Lymphoma ASH Annual Meeting Abstracts 2011 118 21 LBA-6

[B28] Williams ME Hong F Kahl BS Gascoyne RD Wagner LI Krauss JC Horning SJ A subgroup analysis of small lymphocytic and marginal zone lymphomas in the Eastern Cooperative Oncology Group protocol E4402 (RESORT): A randomized phase III study comparing two different rituximab dosing strategies for low tumor burden indolent non-Hodgkin lymphoma ASCO Meeting Abstracts 2012 30 15_suppl) 8007

[B29] Brewster M Hurst D Chai A Lee EJ Upadhyaya GH Dakhil SR Pharmacokinetics (PK) and pharmacodynamics (PD) of rituximab administered by faster infusion in patients with previously untreated diffuse large B-cell (DLBCL) or follicular lymphoma (FL) ASCO Meeting Abstracts 2012 30 15_suppl) 6591

[B30] Federico M Luminari S Dondi A Sacchi S Franco V Pileri S Lombardo M Rossi G Arcaini L Chisesi T R-CVP versus R-CHOP versus R-FM as first-line therapy for advanced-stage follicular lymphoma: Final results of FOLL05 trial from the Fondazione Italiana Linfomi (FIL) ASCO Meeting Abstracts 2012 30 15_suppl 8006

[B31] Salles G Seymour JF Offner F Lopez-Guillermo A Belada D Xerri L Feugier P Bouabdallah R Catalano JV Brice P Rituximab maintenance for 2 years in patients with high tumour burden follicular lymphoma responding to rituximab plus chemotherapy (PRIMA): a phase 3, randomised controlled trial Lancet 2011 377 9759 42 51 10.1016/S0140-6736(10)62175-7 21176949

[B32] Rigacci L Puccini B Cortelazzo S Gaidano G Piccin A D'Arco A Freilone R Storti S Orciuolo E Zinzani PL Bendamustine with or without rituximab for the treatment of heavily pretreated non-Hodgkin's lymphoma patients: A multicenter retrospective study on behalf of the Italian Lymphoma Foundation (FIL) Ann Hematol 2012 91 7 1013 1022 10.1007/s00277-012-1422-5 22349722

[B33] Montillo M Is bendamustine an ideal partner for rituximab in the management of relapsed chronic lymphocytic leukemia? Results of a multicenter Phase II trial Expert Rev Hematol 2012 5 1 43 46 10.1586/ehm.11.75 22272704

[B34] Chang JE Kahl BS Bendamustine: more ammunition in the battle against mantle cell lymphoma Leuk Lymphoma 2012 53 7 1249 1250 10.3109/10428194.2011.654342 22220986

[B35] Sanchez-Gonzalez B Penalver FJ Medina A Guillen H Calleja M Gironella M Arranz R Sebastian E de Ona R Canovas A Clinical experience of bendamustine treatment for non-Hodgkin lymphoma and chronic lymphocytic leukemia in Spain Leuk Res 2012 36 6 709 714 10.1016/j.leukres.2011.10.024 22154023

[B36] Magyari F Simon Z Barna S Udvardy M Varoczy L Illes A Successful administration of rituximab-bendamustine regimen in the relapse of Hodgkin lymphoma after autologous hemopoietic stem cell transplantation Hematol Oncol 2012 30 2 98 100 10.1002/hon.1004 22034208

[B37] Rummel MJ Niederle N Maschmeyer G Banat AG von Gruenhagen U Losem C Kofahl-Krause D Heil G Welslau M Balser C Bendamustine plus rituximab (B-R) versus CHOP plus rituximab (CHOP-R) as first-line treatment in patients with indolent and mantle cell lymphomas (MCL): Updated results from the StiL NHL1 study ASCO Meeting Abstracts 2012 30 18_suppl) 3

[B38] Buchegger F Antonescu C Helg C Kosinski M Prior JO Delaloye AB Press OW Ketterer N Six of 12 relapsed or refractory indolent lymphoma patients treated 10 years ago with 131I-tositumomab remain in complete remission Journal of nuclear medicine: official publication, Society of Nuclear Medicine 2011 52 6 896 900 10.2967/jnumed.111.087460 21571800

[B39] Buchegger F Antonescu C Delaloye AB Helg C Kovacsovics T Kosinski M Mach JP Ketterer N Long-term complete responses after 131I-tositumomab therapy for relapsed or refractory indolent non-Hodgkin's lymphoma Br J Cancer 2006 94 12 1770 1776 10.1038/sj.bjc.6603166 16685263 PMC2361356

[B40] Davies AJ Rohatiner AZ Howell S Britton KE Owens SE Micallef IN Deakin DP Carrington BM Lawrance JA Vinnicombe S Tositumomab and iodine I 131 tositumomab for recurrent indolent and transformed B-cell non-Hodgkin's lymphoma Journal of clinical oncology: official journal of the American Society of Clinical Oncology 2004 22 8 1469 1479 10.1200/JCO.2004.06.055 15084620

[B41] Friedberg JW Fisher RI Iodine-131 tositumomab (Bexxar): radioimmunoconjugate therapy for indolent and transformed B-cell non-Hodgkin's lymphoma Expert Rev Anticancer Ther 2004 4 1 18 26 10.1586/14737140.4.1.18 14748653

[B42] Esmaeli B McLaughlin P Pro B Samaniego F Gayed I Hagemeister F Romaguera J Cabanillas F Neelapu SS Banay R Prospective trial of targeted radioimmunotherapy with Y-90 ibritumomab tiuxetan (Zevalin) for front-line treatment of early-stage extranodal indolent ocular adnexal lymphoma Annals of oncology: official journal of the European Society for Medical Oncology / ESMO 2009 20 4 709 714 10.1093/annonc/mdn692 19150940

[B43] Delaloye AB Antonescu C Louton T Kuhlmann J Hagenbeek A Dosimetry of 90Y-ibritumomab tiuxetan as consolidation of first remission in advanced-stage follicular lymphoma: results from the international phase 3 first-line indolent trial Journal of nuclear medicine: official publication, Society of Nuclear Medicine 2009 50 11 1837 1843 10.2967/jnumed.109.067587 19837764

[B44] Tobinai K Watanabe T Ogura M Morishima Y Hotta T Ishizawa K Itoh K Okamoto S Taniwaki M Tsukamoto N Japanese phase II study of 90Y-ibritumomab tiuxetan in patients with relapsed or refractory indolent B-cell lymphoma Cancer Sci 2009 100 1 158 164 10.1111/j.1349-7006.2008.00999.x 19018755

[B45] Zinzani PL Tani M Fanti S Stefoni V Musuraca G Vitolo U Perrotti A Fina M Derenzini E Baccarani M A phase 2 trial of fludarabine and mitoxantrone chemotherapy followed by yttrium-90 ibritumomab tiuxetan for patients with previously untreated, indolent, nonfollicular, non-Hodgkin lymphoma Cancer 2008 112 4 856 862 10.1002/cncr.23236 18189293

[B46] Press OW Unger JM LeBlanc ML Rimsza LM Friedberg JW Czuczman MS Kaminski MS Braziel RM Spier CM Maloney DG A phase III randomized intergroup trial (S0016) comparing CHOP plus rituximab with CHOP plus iodine-131-tositumomab for front-line treatment of follicular lymphoma: Results of subset analyses and a comparison of prognostic models ASCO Meeting Abstracts 2012 30 15_suppl 8001

[B47] Corazzelli G Frigeri F Marcacci G Capobianco G Arcamone M Becchimanzi C Russo F Pinto A Rituximab plus gemcitabine, ifosfamide, oxaliplatin (R-GIFOX) as salvage therapy for recurrent Hodgkin lymphoma ASCO Meeting Abstracts 2009 27 15S 8579

[B48] El Gnaoui T Dupuis J Belhadj K Rahmouni A Copie-Bergman C Gaillard I Divine M Tabah-Fisch IM Reyes F Haioun C Rituximab, gemcitabine and oxaliplatin (R-GEMOX): An effective regimen for relapsed and refractory B-cell lymphoma ASCO Meeting Abstracts 2006 24 18_suppl) 7562 10.1093/annonc/mdm133 17496309

[B49] Tsimberidou AM Wierda WG Badoux X Wen S Plunkett W O'Brien SM Kipps TJ Jones JA Kantarjian H Keating MJ Evaluation of oxaliplatin, fludarabine, cytarabine, and rituximab (OFAR) combination therapy in aggressive chronic lymphocytic leukemia (CLL) and Richter's syndrome (RS) ASCO Meeting Abstracts 2010 28 15_suppl 6521

[B50] Tsimberidou AM Wierda WG Plunkett WK O'Brien S Lerner S Smith SC Kantarjian HM Keating MJ hase I/II study of oxaliplatin, fludarabine, cytarabine, and rituximab in patients (OFAR2) with Richter's syndrome (RS), and relapsed or refractory B-cell chronic lymphocytic leukemia (CLL) ASCO Meeting Abstracts 2009 27 15S) 7031

[B51] Crescentini RM Sweet KL Liu J Liboy I Dalia S Chavez JC Bello CM Sokol L Sotomayor EM Cabanillas F An update on gemcitabine, rituximab, and oxaliplatin in combination for relapsed/refractory non-Hodgkin lymphomas ASCO Meeting Abstracts 2012 30 15_suppl) 8084

[B52] Fremin C Meloche S From basic research to clinical development of MEK1/2 inhibitors for cancer therapy J Hematol Oncol 2010 3 1 8 10.1186/1756-8722-3-8 20149254 PMC2830959

[B53] Yuan Y Liao YM Hsueh CT Mirshahidi HR Novel targeted therapeutics: inhibitors of MDM2, ALK and PARP J Hematol Oncol 2011 4 16 10.1186/1756-8722-4-16 21504625 PMC3103487

[B54] Ge X Wang X Role of Wnt canonical pathway in hematological malignancies J Hematol Oncol 2010 3 1 33 10.1186/1756-8722-3-33 20843302 PMC2954871

[B55] Elbaz H Stueckle T Tse W Rojanasakul Y Dinu C Digitoxin and its analogs as novel cancer therapeutics Experimental Hematology & Oncology 2012 1 1 4 10.1186/2162-3619-1-4 PMC3506989 23210930

[B56] Brown JR Sharman JP Harb WA Kelly KR Schreeder MT Sweetenham JW Barr PM Foran JM Gabrilove JL Kipps TJ Phase Ib trial of AVL-292, a covalent inhibitor of Bruton's tyrosine kinase (Btk), in chronic lymphocytic leukemia (CLL) and B-non-Hodgkin lymphoma (B-NHL) ASCO Meeting Abstracts 2012 30 15_suppl) 8032

[B57] Byrd JC Furman RR Coutre SE Burger JA Blum KA Sharman JP Flinn IW Grant BW Heerema NA Johnson AJ The Bruton's tyrosine kinase (BTK) inhibitor PCI-32765 (P) in treatment-naive (TN) chronic lymphocytic leukemia (CLL) patients (pts): Interim results of a phase Ib/II study ASCO Meeting Abstracts 2012 30 15_suppl 6507

[B58] Jaglowski SM Jones JA Flynn JM Andritsos LA Maddocks KJ Blum KA Grever MR Geyer SM Woyach JA Johnson AJ A phase Ib/II study evaluating activity and tolerability of BTK inhibitor PCI-32765 and ofatumumab in patients with chronic lymphocytic leukemia/small lymphocytic lymphoma (CLL/SLL) and related diseases ASCO Meeting Abstracts 2012 30 15_suppl 6508

[B59] O'Brien SM Barrientos JC Flinn IW Barr PM Burger JA Navarro T James DF Hedrick E Friedberg JW Brown JR Combination of the Bruton's tyrosine kinase (BTK) inhibitor PCI-32765 with bendamustine (B)/rituximab (R) (BR) in patients (pts) with relapsed/refractory (R/R) chronic lymphocytic leukemia (CLL): Interim results of a phase Ib/II study ASCO Meeting Abstracts 2012 30 15_suppl 6515

